# Relationship between Job Burnout, Depressive Symptoms, and Career Choice Regret among Chinese Postgraduates of Stomatology

**DOI:** 10.3390/ijerph192316042

**Published:** 2022-11-30

**Authors:** Lu Yang, Li Yan, Xiaogang Zhong, Huiqing Long, Fangchun Chen, Xin Jin

**Affiliations:** 1 Key Laboratory of Psychoseomadsy, Stomatological Hospital of Chongqing Medical University, Chongqing 401147, China; 2 Chongqing Key Laboratory of Oral Diseases and Biomedical Sciences, Chongqing 401147, China; 3 School of Public Health and Management, Chongqing Medical University, Chongqing 400016, China; 4 NHC Key Laboratory of Diagnosis and Treatment on Brain Functional Diseases, The First Affiliated Hospital of Chongqing Medical University, Chongqing 400016, China

**Keywords:** job burnout, depressive symptoms, career choice regret, stomatology, postgraduates, influencing factors

## Abstract

A qualified Chinese dental postgraduate requires at least eight years of training. The huge academic burden, strict clinical requirements, and high workload increases the risk of job burnout, depression symptoms, and career choice regret of dental postgraduates, which may cause one to waver in their choice of a career as a doctor. Therefore, we aimed at assessing the relationship between job burnout, depressive symptoms, and career choice regret among Chinese dental postgraduates. The Chongqing Stomatological Association conducted an online cross-sectional study among 558 dental postgraduates in China, with an average age of 22.54 ± 2.44. Demographic information, the Maslach Burnout Inventory, the 2-item Primary Care Evaluation of Mental Disorders scale, and career choice regret scale were included in the questionnaire. About 41.0% of dental postgraduates experienced job burnout, 44.1% had depressive symptoms, and 41.6% reported career choice regret. Logistic regression analysis indicated the risk factors for job burnout were time worked/studied per week, depressive symptoms, and career choice regret. Job burnout and career choice regret was significantly related to depressive symptoms (*p* < 0.001). Risk factors for career choice regret were gender, postgraduate entrance examination score, daily hours of sleep, job burnout, and depressive symptoms. Such results suggest that job burnout, depressive symptoms, and career choice regrets are prevalent among dental postgraduates. Accurate measures should be taken to change this situation.

## 1. Introduction

According to the data published in the Chinese Health Statistics Yearbook, the number of registered dentists in China was 221,000 in the 2020. The World Health Organization recommends a dentist to population ratio of 1:5000. China’s population of 1.41 billion requires at least 282,000 dentists [1]. A qualified Chinese medical postgraduate requires at least eight years of training, during which they face an abundance of academic challenges, strict clinical requirements, and severe life pressures. With regards to the academic aspect, apart from studying heavy courses, there is the added pressure of strict examinations and graduation papers. In the clinical aspect, in addition to dealing with some intractable cases, they also need to learn specific practical operations to improve patients’ sense of trust. In terms of personal/social life, the shrinking social circle, and the inability to be financially independent are challenges for postgraduates in China. These reasons are contributing factors to the risk of dropping out of graduate medical school [2]. After graduation from medical colleges, they are faced with a huge workload, academic pressure, and promotion challenges, which increase the risk of burnout, depressive symptoms, and career choice regret [2,3,4,5].

Burnout is a prolonged response to interpersonal stressors and chronic emotions on the job [6]. It is characterized by high levels of emotional exhaustion, depersonalization, and a low sense of personal accomplishment [7]. A study performed in the United States of America (USA) found that 52.8% of medical students had experienced burnout [8], and this number was 50% in Brazil [9]. In Chinese neurology postgraduates, burnout incidences were reported to be as high as 83.6% [10]. Burnout is widespread among medical students [8]. Burnout is widespread among medical students [11]. According to previous studies, gender is one of the risk factors for burnout. Compared with women, the men are not prone to expressing emotions, so they always take a negative attitude towards difficulties and are more easily affected by unhealthy emotions [12]. Additionally, younger age, long working hours, and higher study year were associated with increased job burnout levels for medical postgraduate students [13,14]. The survey on Chinese postgraduates of neurology also showed that entrance examination score of postgraduate and status of marriage were closely related to burnout for postgraduate students [10]. A study covering 43 countries reported that the prevalence of depressive symptoms in medical students was 27.7% [15]. Depressive symptoms in dental students have been associated with financial insecurity and low satisfaction with peers and teachers [16,17]. Other studies also indicated that senior students and short sleep time were associated with increased depressive symptoms levels for medical graduate students [18]. Career choices of Chinese dental students were influenced by economy and prestige [19]. The numerous demographic characteristics can affect job burnout, depressive symptoms, and career choice regret levels for medical postgraduates.

Burnout is closely associated with depression [8,20]. Medical students who experience high burnout levels also exhibit high levels of depressive symptoms [21,22,23]. Depression symptoms include low mood, anhedonia, and fatigue. These symptoms overlap with burnout [24]. Previous studies indicated that burnout and depressive symptoms are influenced by some common factors, including work stress. These factors have been shown to have an obvious overlap. Therefore, burnout may be a pre-depression state [25,26]. Furthermore, burnout symptoms are consistent with depressive symptoms [27].

Burnout is also associated with career choice regret [28,29]. In the USA, it has been pointed out that burnout is the single greatest predictor of career choice regret among resident physicians [30]. Burnout reduces clinical effectiveness and job satisfaction, which leads to career choice regret [28]. On the contrary, a study from China reported that career choice regret is the strongest risk factor for burnout among Chinese neurology postgraduates [10]. The possible explanation was that career choice regret may lead to a stronger dislike of the occupation, which increases burnout risk [31].

With the outbreak of Coronavirus disease 2019 (COVID-19), the workload for healthcare workers markedly increased. Medical postgraduates, the core of a country’s medical workforce in the coming decades, are also subjected to high workloads, and their burnout and mental health status are causes for clinical concern, especially dental postgraduates, as they regularly have face-to-face contact with patients’ saliva, blood, and crevicular gingival fluid. The aerosol-filled work environment exposes them to high risks of infection. Therefore, understanding job burnout, depressive symptoms, and career choice regret among dental postgraduates is of great importance in dental education.

A limited number of studies have investigated burnout, depressive symptoms, and career choice regret among dental postgraduates in China, especially during the COVID-19 pandemic. We aimed at assessing the prevalence of burnout, depressive symptoms, and career choice regret among Chinese dental postgraduates to determine associated factors. Our findings provide reference for research and implementation of follow-up intervention measures. We suggest the following hypotheses: (1) postgraduates of stomatology have a different degree of work burnout, depressive symptoms, and career choice regret; (2) work burnout, depressive symptoms, and career choice regret could be affected by numerous demographic characteristics; (3) work burnout, depressive symptoms, and career choice regret are closely associated with each other.

## 2. Methods

### 2.1. Participants

The Chongqing Stomatological Association conducted this cross-sectional survey between February 2021 to March 2021. First, the staff of Chongqing Stomatological Association contacted the directors in charge of the college of stomatology or dental departments in local hospitals with dental postgraduates via WeChat (free information and telephone application), telephone, or e-mail. If the directors agreed to take part in this study, postgraduates of stomatology were invited to participate in this study. The questionnaire was constructed based on Questionnaire Star (https://www.wjx.cn/, accessed on 1 February 2021), a professional online questionnaire survey platform in China. Through this platform, questionnaire contents were transformed into a link, which was sent to the directors that agreed to participate in this survey. Then, the directors forwarded the link to the postgraduates’ WeChat group (a communication tool similar to chat room). The front page of the questionnaire introduced the background and purpose of the survey, informed participants of their rights and potential risks, and informed them that the survey was voluntary and anonymous. Consent was assumed for any participant who returned a completed survey.

### 2.2. Quality Control

To ensure the accuracy of the returned questionnaire, some settings were made as follows: submission was only successful after all questions had been completed, otherwise there were reminders that the questionnaire was incomplete and could not be submitted. If two or more consecutive questionnaires are from the same hospital or university, only one questionnaire will be included, while the other identical questionnaire will be excluded. Furthermore, to ensure the uniqueness of the questionnaire, each internet protocol address could only be submitted once. This study was reviewed and approved by the Ethical Committee of the Stomatological Hospital of Chongqing Medical University.

### 2.3. Research Instruments

The questionnaire was self-administrated. Each participant was given a four-part questionnaire. The first part had demographic characteristics, including participants’ gender, academic year, degree type, family income, postgraduate entrance examination score, worked/studied time per week, daily hours of sleep, whether they had children, marital status, and whether they had ever engaged in a part-time job.

In the second part, we evaluated job burnout among participants through the Maslach Burnout Inventory [32]. The 22-item scale evaluates the three dimensions (emotional exhaustion, depersonalization, and personal accomplishment) of job burnout. The three dimensions contain 9, 5, and 8 items, respectively. All items are scored from 0 to 6, and the total score for each dimension is calculated by adding them up. Emotional exhaustion with a score of ≥27, or depersonalization with a score of ≥10, indicates a high degree of job burnout [21], and it has been successfully used among Chinese populations [10,32]. Cronbach’s α coefficient of the Maslach Burnout Inventury-22 was 0.704, with emotional exhaustion being 0.904, personal accomplishment being 0.862, and depersonalization being 0.613 in this study.

In the third part, we measured respondents’ depressive symptoms using the 2-item Primary Care Evaluation of Mental Disorders (PRIME-MD), which has been widely used in previous studies [9]. Respondents who answered “yes” to at least one of the following two questions were considered to have depressive symptoms, “Have you often been bothered by feeling down, depressed, or hopeless during the past month?” and “Have you often been bothered by having little interest or pleasure in doing things during the past month?”. The PRIME-MD is similar to validated instruments in assessing depressive symptoms and has been shown to have a good sensitivity and specificity [33,34]. Cronbach’s α coefficient of PRIME-MD was 0.870 in this study.

Finally, career choice regret was evaluated using the question: “If you could come back, would you still choose to study medicine again?”. The answers included “Yes, Not sure and No”. “No” in this case implied career choice regret. This method of assessing career choice regret has been widely used in previous studies [10,35].

### 2.4. Statistical Analysis

SPSS 21.0 (IBM Corp, Armonk, NY, USA) was used for all analyses. Categorical variables are expressed as frequencies and percentages, while quantitative variables are expressed as mean ± standard deviation. Univariate analysis was conducted by chi-square tests or the Fishers’ exact test. Binary logistic regression models (enter model, backward elimination model, and forward elimination model) were used to evaluate the potential influencing factors. We fitted two logistic regression models. Model one included the full range of respondent demographic characteristics, while model two was further adjusted for job burnout, depressive symptoms, and career choice regret. When the variance inflation factor (VIF) value in the regression model exceeded 10, multi-collinearity was considered between the variables, which were then removed from the model. *p* < 0.05 was the threshold for statistical significance.

## 3. Results

Dental postgraduates from the five provinces of western China participated in this survey. A total of 580 questionnaires were returned, and 22 questionnaires were excluded for the same answer for the whole questionnaire, leaving 558 questionnaires. Valid questionnaires were from the five provinces as follows: Chongqing (268, 48.0%), Sichuan (134, 24.0%), Guizhou (117, 21.0%), Gansu (26, 4.7%), and Yunnan (13, 2.3%).

Overall, 69.2% of participants were female, 93.9% were pursuing master’s degrees, 70.8% were engaged in clinical practice, 36.2% reported family income less than 5000 CNY per month, 30.1% reported postgraduate entrance examination scores less than 330 (higher scores can give preference to supervisor), 35.8% worked for more than 55 h per week, 78.7% slept for 6–8 h per day, 92.1% were unmarried, 94.4% had no children, and 86.6% had not participated in part-time jobs (Table 1).

**Figure 1:**
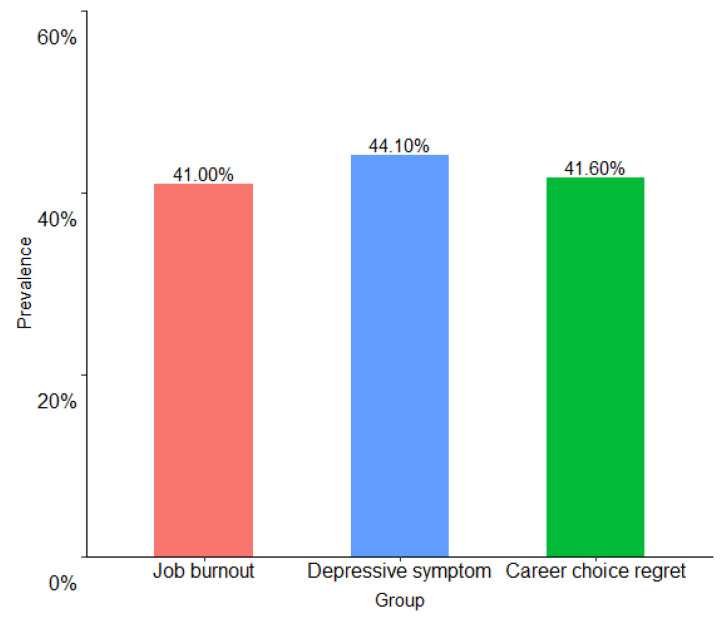
Prevalence of job burnout, depressive symptoms, and career choice regret.

Incidences of job burnout, depressive symptoms, and career choice regret among dental postgraduates exceeded 40%. Specifically, 41.0% reported job burnout, 44.1% had depressive symptoms, and 41.6% reported the career choice regret (Table 2, Figure 1).

Univariate analysis showed that the factors associated with job burnout included the time worked/studied per week (*p* = 0.011) and daily hours of sleep (*p* < 0.001). With regards to depressive symptoms, the factors were daily hours of sleep (*p =* 0.012). Regarding career choice regret, the factors were gender (*p* = 0.007), postgraduate entrance examination score (*p* = 0.017), and daily hours of sleep (*p* < 0.001). The daily hours of sleep were the common influencing factor for job burnout, depressive symptoms, and career choice regret (Table 3).

In model one, only demographic characteristics were included in the regression analysis. It was found that the factors associated with job burnout included daily hours of sleep (*p* < 0.001) and whether participants had children (*p* = 0.044). The factor associated with depressive symptoms was daily hours of sleep (*p* = 0.013). The factors associated with career choice regret were gender (*p* = 0.004), postgraduate entrance examination score (*p* = 0.008), and daily hours of sleep (*p* < 0.001) (Table 4).

**Figure 2:**
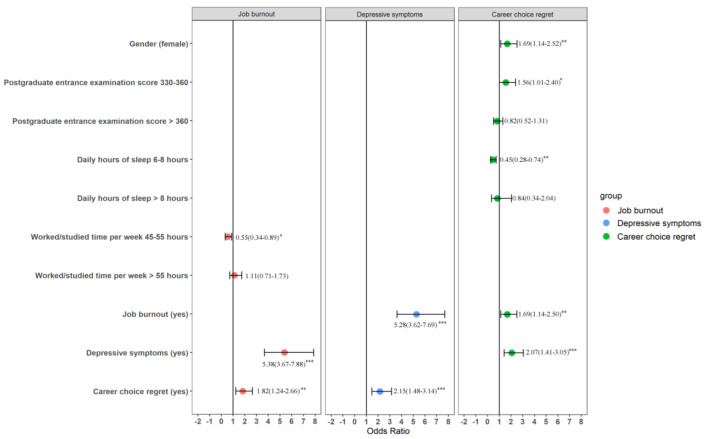
Risk factors of burnout, career choice regret, and depressive symptoms in dental postgraduates. * *p* < 0.05, ** *p* < 0.01, *** *p* < 0.001.

In model two, job burnout, depressive symptoms, and career choice regret were included based on model one. Work/study time per week (*p* = 0.009), depressive symptoms (*p* < 0.001), and career choice regret (*p* = 0.002) were associated with job burnout. Job burnout (*p* < 0.001) and career choice regret (*p* < 0.001) were associated with depressive symptoms. Gender (*p* = 0.009), postgraduate entrance examination score (*p* = 0.011), daily hours of sleep (*p* = 0.003), job burnout (*p* = 0.009), and depressive symptoms (*p* < 0.001) were associated with career choice regret (Table 5, Figure 2).

## 4. Discussion

We used an online self-administrated questionnaire to investigate the job burnout, depressive symptoms, and career choice regret of Chinese dental postgraduates. The prevalence and the influencing factors of job burnout, depressive symptoms, and career choice regret were explored. To our knowledge, this is a large-scale survey of dental postgraduate in China. 

### 4.1. Prevalence and Factors of Job Burnout

In this study, we found that the prevalence of job burnout among Chinese dental postgraduates was 41.0%, which was higher than those of dental students in Saudi Arabia (30.1%) [36], Mexico (17.8%) [37], and Spain (26%) [38]. The possible reason is that Chinese postgraduates have to concern themselves with their grade point average (GPA) and also need to focus on clinical work and scientific research training to meet graduation requirements [39,40]. Academic workload plays an important role in the development of job burnout, and it is a starting factor for determining the burnout period [41,42]. Job burnout generally increases as the course progresses [13]. The job burnout of Chinese dental postgraduates is lower than the neurology postgraduates (83.6%), possibly because of the complexity of diagnosis and treatment of related diseases in neurology. We found that daily hours of sleep and whether participants had children influenced job burnout. Dental postgraduates with 6–8 h of sleep had the lowest risk of burnout. This may be because insufficient sleep reduces work efficiency, resulting in reduced sense of satisfaction and achievement [43,44]. Dental postgraduates with children had a lower risk of job burnout, probably because children bring happiness to parents [45]. Having children has been shown to mitigate psychological burnout [46]. After adjustment for depressive symptoms and career choice regret, we found that daily hours of sleep and whether participants had children in model two had no statistical significance for job burnout. Depressive symptoms and career choice regret were risk factors for job burnout. Previous studies have shown that symptoms and related factors for job burnout and depression exhibit obvious overlaps [26,45]. We established that job burnout is strongly associated with career and specialty choice regret, consistent with previous studies [47]. Therefore, attention should be paid to job burnout among postgraduates to promote their physical and mental health. Moreover, we found that work/study time per week influenced burnout outcomes. Working too many hours may increase fatigue and decrease career satisfaction, which may increase job burnout risks [48,49]. Moderate working time helps dental postgraduates to complete their studies better and have adequate rest time to avoid exhaustion and reduce burnout risks.

### 4.2. Prevalence and Factors of Depressive Symptoms

The prevalence of depressive symptoms among Chinese dental postgraduates was 44.1%, which is higher than that of Australian dental postgraduates (24%) [50] and close to that of Saudi Arabian dental postgraduates (42.2%) [51]. This suggests that dental postgraduates in different countries have different levels of depressive symptoms, which may be caused by the differences between medical settings and educational environment [40,52,53]. Our multivariate analysis showed that daily hours of sleep influences depressive symptoms. Sleep time was associated with depressive symptoms, consistent with findings from previous studies [54]. Chronic sleep deprivation leads to circadian rhythm disorders and elevated cortisol levels, resulting in depression [55]. We found that daily hours of sleep were not significant for depressive symptoms in model two. Meanwhile, job burnout was a risk factor for depressive symptoms. There is a significant correlation between job burnout and depressive symptoms, and depressive symptoms were also a risk factor for high intention to leave [10,56,57]. Depressive symptoms and career choice regret are predictors for job burnout [10,56].

### 4.3. Prevalence and Factors of Career Choice Regret

The prevalence of career choice regret among Chinese dental postgraduates was 41.6%, which is lower than that of Chinese neurology postgraduates (46.6%) [10] and emergency medicine students in Turkey (67.4%) [58]. This is probably due to the heavy clinical workload of these two medical specialties, leading to higher regrets of career choice [10,58]. Multivariate analysis showed that gender, postgraduate entrance examination scores, and daily hours of sleep influence career choice regret. Career choice regret incidences are higher in females than in males. This can be explained by the “double burden” of household and working duties of females. Usually, females spend more time on household chores after work, leaving insufficient recovery time [59]. Postgraduate entrance examination scores between 330–360 had the highest risk of career choice regret. Since the scores affect the process of choosing a mentor, students with scores in the 330–336 (middle) range may have higher expectations for this process but get results that fall short or worse, increasing their career choice regret. Dental postgraduates with 6–8 h of sleep had the lowest risk of career choice regret. This could have been because adequate sleep time leads to a better mental state, improved work efficiency, and satisfaction. After adjusting for job burnout and depressive symptoms, we found that gender, postgraduate entrance examination scores, and daily hours of sleep were still left in model two.

Interestingly, we found that job burnout, depressive symptoms, and career choice regret were closely associated. Job burnout affects physical and mental health and causes higher degrees of depressive symptoms [60]. Severe job burnout can lead to negative work attitudes and reduced work efficiency [61], which may increase the risk of career choice regret. Moreover, career choice regret plays a mediating role in depression development [62].

### 4.4. Further Measures

Dental training courses require students to master not only theory, but also clinical proficiency and patient relationship, affecting one’s physical and mental wellbeing [63]. Therefore, we should pay more attention to the physical and mental health of dental students. Prevention of job burnout, depressive symptoms, and reduction of career choice regret are important for ensuring a healthy life and study outcomes for dental graduate students in China. This requires joint efforts from the government, hospitals, and students. First, the government should integrate clinical tasks and course studies for dental graduate students to reduce the course burden. Second, emotional intelligence theory suggests that emotional regulation skills help reduce or adjust to the bad emotions in oneself and others by maintaining emotions [64]. Hospitals should increase students’ physical and mental health education courses and provide long-term career planning routes. Third, it is important to maintain a good mindset, along with a clear understanding of burnout, depressive symptoms, and career choice regrets.

## 5. Limitations and Future Research

This study is subject to certain limitations. Firstly, dental postgraduates who participated in this study came from the five provinces of western China. The sample did not cover all provinces of China, and the sample size was not very large. Therefore, we should be cautious when assigning these results to the profiles of all dental postgraduates. Secondly, this study adopted a cross-sectional sampling, so we cannot examine the dynamic changes of job burnout, depressive symptoms, and career choice regret to determine the causal relationships. Thirdly, during COVID-19, a certain impact can be brought to the mental health of postgraduates, but the analysis in this aspect has not been conducted. Fourthly, dental postgraduates are mainly divided into clinical practice and academic practice for discussion. However, students from different oral sub majors (including prosthodontics, oral and maxillofacial surgery, and endodontics, etc.) may have different pressures in learning and clinical practice, which should be analyzed as a key point in our future research to attain a more detailed understanding of their situation. Fifthly, it is undeniable that dental postgraduate will also face pressures after graduation, such as employment pressure, doctor-patient relationship pressure, and economic pressure. As a result, it is also indispensable to have a clear picture in this aspect for better evaluation and adjustments on their psychology. Therefore, a larger sample size and more targeted scale design are needed for future research so as to collect more comprehensive data and lay the foundation for more accurate analysis.

## 6. Conclusions

Job burnout, depressive symptoms, and career choice regrets are prevalent among dental postgraduates, and these factors are closely associated with each other. To reduce the influence of these adverse factors on postgraduates of stomatology, the education about the physiological and psychological symptoms of job burnout and depression should be conducted for postgraduates. The strategies to prevent and manage pressure should be found to improve the well-being of postgraduates. In this way, the quantity and quality of dentists in the future can be guaranteed.

## Figures and Tables

**Table 1:** Demographic characteristics of the participants.

Variables	Number (*n*)	Percent (%)
Gender		
Male	172	30.8
Female	386	69.2
Academic year		
First-year, master’s degree	215	38.5
Second-year, master’s degree	146	26.2
Third-year, master’s degree	163	29.2
Doctor’s degree	34	6.1
Degree type		
Clinical practice	395	70.8
Academic practice	163	29.2
Family income (CNY per month)		
<5000	202	36.2
5000–10,000	218	39.1
>10,000	138	24.7
Postgraduate entrance examination score		
<330	168	30.1
330–360	221	39.6
>360	169	30.3
Worked/studied time per week (hours)		
<45	188	33.7
45–55	170	30.5
>55	200	35.8
Daily hours of sleep		
<6	91	16.3
6–8	439	78.7
>8	28	5.0
Marital status		
Signal	287	51.4
Partner	227	40.7
Married	44	7.9
Whether have children		
No	527	94.4
Yes	31	5.6
Who had ever undertaken part-time job		
No	483	86.6
Yes	75	13.4

*n*: number of the participants. %: proportion of the participants.

**Table 2:** Prevalence of job burnout, depressive symptoms, and career choice regret.

Variables	Number (*n*)	Percent (%)
Job burnout		
Yes	229	41.0
No	329	59.0
Depressive symptoms		
Yes	246	441
No	312	55.9
Career choice regret		
Yes	232	41.6
No	326	58.4

*n*: number of participants; %: proportion of participants.

**Table 3:** Univariate analysis of job burnout, depressive symptoms, and career choice regret.

Variables	Job Burnout	Depressive Symptoms	Career Choice Regret
Gender			
Male	65(37.8%)	70(40.7%)	57(33.1%)
Female	164(42.5%)	176(45.6%)	175(45.3%)
χ^2^	1.085	1.158	7.287
*p* value	0.298	0.282	0.007
Academic year			
First-year, master’s degree	85(35.9%)	83(38.6%)	82(38.1%)
Second-year, master’s degree	61(41.8%)	70(47.9%)	64(43.8%)
Third-year, master’s degree	67(41.1%)	77(47.2%)	68(41.7%)
Doctor’s degree	16(47.1%)	16(47.1%)	18(52.9%)
χ^2^	0.744	4.282	3.161
*p* value	0.863	0.233	0.367
Degree type			
Clinical practice	165(41.8%)	171(43.3%)	161(40.8%)
Academic practice	64(39.3%)	75(46.0%)	71(43.6%)
χ^2^	0.300	0.347	0.372
*p* value	0.584	0.556	0.542
Family income (CNY per month)			
<5000	79(39.1%)	87(43.1%)	93(46.0%)
5000–10,000	98(45.0%)	106(48.6%)	85(39.0%)
>10,000	52(37.7%)	53(38.4%)	54(39.1%)
χ^2^	2.335	3.712	2.596
*p* value	0.311	0.156	0.273
Postgraduate entrance examination score			
<330	71(42.3%)	77(45.8%)	67(39.9%)
330–360	89(40.3%)	101(45.7%)	107(48.4%)
>360	69(40.8%)	68(40.2%)	58(34.3%)
χ^2^	0.161	1.458	8.119
*p* value	0.923	0.482	0.017
Worked/studied time per week (hours)			
<45	82(43.6%)	83(44.1%)	77(41.0%)
45–55	54(31.8%)	72(42.4%)	70(41.2%)
>55	93(46.5%)	91(45.5%)	85(42.5%)
χ^2^	9.024	0.370	0.111
*p* value	0.011	0.831	0.946
Daily hours of sleep			
<6	55(60.4%)	53(58.2%)	54(59.3%)
6–8	163(37.1%)	182(41.5%)	164(37.4%)
>8	11(39.3%)	11(39.3%)	14(50.0%)
χ^2^	16.963	8.889	15.857
*p* value	*p* < 0.001	0.012	*p* < 0.001
Marital status			
Signal	122(42.5%)	133(46.3%)	116(40.4%)
Partner	90(39.6%)	96(42.3%)	97(42.7%)
Married	17(38.6%)	17(38.6%)	19(43.2%)
χ^2^	0.543	1.419	0.330
*p* value	0.762	0.492	0.848
Whether have children			
No	221(41.9%)	235(44.6%)	220(41.7%)
Yes	8(25.8%)	11(35.5%)	12(38.7%)
χ^2^	3.148	0.985	0.111
*p* value	0.076	0.321	0.739
Who had ever undertaken part-time job			
No	194(40.2%)	212(43.9%)	199(41.2%)
Yes	35(46.7%)	34(45.3%)	33(44.0%)
χ^2^	1.134	0.055	0.209
*p* value	0.287	0.815	0.647

**Table 4:** Regression model one of job burnout, depressive symptoms, and career choice regret.

Variables	Job Burnout		Depressive Symptoms		Career Choice Regret	
	OR (95%CI)	*p*	OR (95%CI)	*p*	OR (95%CI)	*p*
Gender (female)					1.76 (1.20–2.59)	0.004
Postgraduate entrance examination score						0.008
<330					1 (Reference)	
330–360					1.53 (1.00–2.32)	0.048
>360					0.80 (0.50–1.25)	0.323
Daily hours of sleep		<0.001		0.013		<0.001
<6	1 (Reference)		1 (Reference)		1 (Reference)	
6–8	0.37 (0.23–0.59)	<0.001	0.51 (0.32–0.80)	0.004	0.37 (0.23–0.60)	<0.001
>8	0.40 (0.17–0.96)	0.41	0.46 (0.20–1.10)	0.082	0.66 (0.28–1.59)	0.357
Whether have children (yes)	0.42 (0.18–0.98)	0.044				

**Table 5:** Regression model two of job burnout, depressive symptoms, and career choice regret.

Variables	Job Burnout		Depressive Symptoms		Career Choice Regret	
	OR (95%CI)	*p*	OR (95%CI)	*p*	OR (95%CI)	*p*
Gender (female)					1.69(1.14–2.52)	0.009
Postgraduate entrance examination score						0.011
<330					1 (Reference)	
330–360					1.56 (1.01–2.40)	0.045
>360					0.82 (0.52–1.31)	0.414
Daily hours of sleep						0.003
<6					1 (Reference)	
6–8					0.45 (0.28–0.74)	0.002
>8					0.84 (0.34–2.04)	0.694
Worked/studied time per week (hours)		0.009				
<45	1 (Reference)					
45–55	0.55 (0.34–0.89)	0.015				
>55	1.11 (0.71–1.73)	0.637				
Job burnout (yes)			5.28 (3.62–7.69)	<0.001	1.69 (1.14–2.50)	0.009
Depressive symptoms (yes)	5.38 (3.67–7.88)	<0.001			2.07 (1.41–3.05)	<0.001
Career choice regret (yes)	1.82 (1.24–2.66)	0.002	2.15 (1.48–3.14)	<0.001		

## Data Availability

The datasets used in this study are available in the manuscript, further requests should be made by directly contacting the corresponding author.
